# Concrete vs. Abstract Semantics: From Mental Representations to Functional Brain Mapping

**DOI:** 10.3389/fnhum.2019.00267

**Published:** 2019-08-02

**Authors:** Nadezhda Mkrtychian, Evgeny Blagovechtchenski, Diana Kurmakaeva, Daria Gnedykh, Svetlana Kostromina, Yury Shtyrov

**Affiliations:** ^1^Laboratory of Behavioral Neurodynamics, St. Petersburg State University, Saint Petersburg, Russia; ^2^Department of Clinical Medicine, Center of Functionally Integrative Neuroscience (CFIN), Aarhus University, Aarhus, Denmark

**Keywords:** concrete and abstract semantics, concreteness effect, mental representation, brain, memory trace, psycholinguistics, functional brain mapping

## Abstract

The nature of abstract and concrete semantics and differences between them have remained a debated issue in psycholinguistic and cognitive studies for decades. Most of the available behavioral and neuroimaging studies reveal distinctions between these two types of semantics, typically associated with a so-called “concreteness effect.” Many attempts have been made to explain these differences using various approaches, from purely theoretical linguistic and cognitive frameworks to neuroimaging experiments. In this brief overview, we will try to provide a snapshot of these diverse views and relationships between them and highlight the crucial issues preventing this problem from being solved. We will argue that one potentially beneficial way forward is to identify the neural mechanisms underpinning acquisition of the different types of semantics (e.g., by using neurostimulation techniques to establish causal relationships), which may help explain the distinctions found between the processing of concrete and abstract semantics.

## Defining Concreteness and Abstractness

One can often encounter in the literature such terms as “concrete and abstract concepts,” “concrete and abstract words,” or “concrete and abstract semantics.” What is the difference? In psycholinguistic and cognitive frameworks, concepts may be termed as the knowledge about a particular category (Barsalou et al., 2003), as a combination of atomic units of information and meaningful relationships between those units (Payne et al., 2007), or as “a mental representation of a class or individual which deals with *what* is being represented and *how* that information is typically used during the categorization” (Smith, 1989, p. 502). Such mental (internal or cognitive) representations (Paivio, 1990) are widely investigated in cognitive psychology, psycholinguistics, philosophy of mind and related fields (Carruthers and Cummins, 1990), but often without a clear connection to neural representations, which are more commonly addressed in brain research, neuroscience and neuroimaging. It is believed that the most important concepts (called lexical concepts) have an expression in the language in the form of individual words (= are labeled by words; Margolis and Laurence, 1999) and are thereby “our representation of word meaning” (Murphy, 2002, p. 392). In this regard, in most concept studies, linguistic stimuli are used and thus the terms “concept,” “word semantics,” and “word meaning” are often used interchangeably. Traditionally, words/concepts are subdivided into concrete and abstract types, and this distinction is considered in many contemporary psycholinguistic and cognitive studies. As often claimed, concrete concepts/words have clear references to material objects (e.g., *dog, house*), whereas references of abstract ones are not physical entities, but more complex mental states (e.g., *thought, happiness*), conditions (*uncertainty*), situations (*encounter*), and relationships (*employment*) (Borghi and Binkofski, 2014). However, even this seemingly simple distinction is not unequivocal. For instance, Myachykov and Fischer (2019) have argued that, in addition to this phenomenological dimension of abstractness, there are also sensorimotor and contextual aspects, and the same word/concept may be both concrete or abstract depending on different dimensions. Sensorimotor and contextual dimensions are, in turn, determined by individual life experience of lexicon acquisition and usage. Therefore, one way to extricate from this tangle could be studying processing of novel words, whose meanings are not yet represented in the participants’ minds. Such an approach may solve the problem of conceptual confusion – the first obstacle to establishing of clear links between theoretical descriptions and the brain mechanisms which underlie representations of these different knowledge types in the brain.

## Theoretical Accounts

Research of concrete and abstract concepts has a long history; a landmark event in its modern period was Paivio’s seminal article “Abstractness, imagery, and meaningfulness in paired-associate learning” (Paivio, 1965). Numerous behavioral experiments using lexical decision, recognition, word naming, and other behavioral tasks demonstrated that concrete concepts, in comparison with abstract ones, are better remembered (Schwanenflugel et al., 1992), recognized (Fliessbach et al., 2006), faster read and comprehended (Schwanenflugel and Shoben, 1983), and faster learnt (Mestres-Missé et al., 2014). Similar results were revealed with respect to the processing of concrete and abstract verbs (Alyahya et al., 2018) and definitions (Borghi and Zarcone, 2016). This advantage of concrete over abstract semantics is usually called “concreteness effect”; to help explain it, Paivio suggested the so-called dual-coding theory (DCT, Paivio, 1990) which posits two functional systems associated with semantic memory: verbal-based and imagery-based (non-verbal). These representational systems are interrelated and can be active independently or in parallel. According to DCT, whereas the verbal system may be responsible for coding both concrete and abstract concepts linguistically, the non-verbal imagery system is primarily involved in coding concrete – but not abstract – concepts, enhancing their processing and leading to behaviorally observed advantages (Kuiper and Paivio, 1977).

Notably, some investigations showed that concrete words elicit faster responses in lexical decision task only when there is no context information helping to understand the meaning; when context is available, the concreteness effect is reduced or absent (Schwanenflugel and Shoben, 1983). These observations were explained by the context-availability theory (CAT), which claims that concrete and abstract concepts have different amount of semantic associations: concrete concepts have stronger associative connections with fewer contexts, while abstract concepts have weaker associative connections with a larger number of contexts. This, in turn, means that providing relevant context information may eliminate the “concreteness effect” leading to equally efficient processing of both semantic types.

A similar view on the distinctions between concrete and abstract words suggests that they are represented in mind in qualitatively different ways (Crutch, 2006). This hypothesis was based on the study of different types of semantic errors in patients with deep dyslexia. According to this account, concrete words have hierarchical semantic structure, which relies on categorical interrelationship (superordinate and co-ordinate), whereas abstract representations have, on the contrary, associative architecture (with connections between words commonly used together).

Other cognitive frameworks, rather than stressing the differences between abstract and concrete processing mechanisms, focus on searching for their similarities. For instance, the embodied cognition view on language grounds semantic representations in bodily functions (perception, action) and proposes that abstract word processing, in the same way as that of concrete words, relies, at least in part, on sensorimotor systems (Glenberg et al., 2008; Pulvermüller, 2013, see Borghi et al., 2017, for review of embodied views on concrete/abstract concepts). Indeed, a comparison of acquisition and processing of abstract semantics in children with typical language development, atypical development, and autism showed no significant differences between these groups, also indicating the absence of specific mechanisms of abstract knowledge acquisition (Vigliocco et al., 2018). This, however, still does not exclude a more substantial contribution of the linguistic system into the abstract processing found in some studies (e.g., Sakreida et al., 2013).

In cognitive linguistics, a somewhat similar approach is offered by the so-called conceptual metaphor theory (CMT), an influential theoretical framework, according to which abstract concepts may be understood in reference to more concrete words by using metaphors (Lakoff and Johnson, 1980). However, in development, metaphors become available later than basic abstract knowledge as such; furthermore, it has been argued that not every abstract concept can be fully understood metaphorically, i.e., in terms of concrete words (Borghi and Zarcone, 2016).

One theoretically contentious issue in accounting for concrete and abstract features of word semantics is that of a relationship between “concreteness” and “emotionality”. Many authors consider words connected to emotions (for example, *love*, *joy, fear*) as a kind of abstract concepts (see, e.g., Dreyer and Pulvermüller, 2018) because they lack specific subject-relatedness. However, consideration of abstractness from embodied, rather than purely phenomenological dimension allows referring to emotions as concrete (embodied in individual experience) items (Myachykov and Fischer, 2019). Furthermore, some authors divide all concepts into three types: concrete, abstract and emotional (Altarriba and Bauer, 2004). This latter approach seems somewhat controversial, as it does not appear to be based on uniform classification criteria. Moreover, both concrete and abstract words may possess less or more emotional meaning (consider, e.g., *joy* vs. *justice*, or *cake* vs. *pencil*); further, this may depend on a person’s individual experience. To put it differently, it is uncertain why, in the Altarriba and Bauer (2004) classification, such words as *win* or *jeopardy* were included into the group of abstract words while *daughter* and *dentist* were treated as concrete words, even though their meaning clearly carries emotional aspects.

Perhaps a more convincing approach links emotional experience with abstract concepts (Kousta et al., 2011). The so-called affective grounding hypothesis (AGH) makes several specific suggestions in this respect (Lenci et al., 2018). First, abstract and concrete concepts differ in the extent of involvement of two types of information: experiential (sensory, motor, and affective) and linguistic (verbal associations); this clearly resonates with Paivio’s dual-coding account. Second, concrete concepts are mainly grounded in sensory-motor information, whereas abstract word meanings are underpinned predominantly by linguistic and emotional information. Finally, the prevalence of these specific types of information plays a crucial role in acquisition as well as further representation of both concrete and abstract concepts (Vigliocco et al., 2009). As a side note, this approach provides a way to define specific semantics as a flexible combination of experiential and linguistic features, suggesting that abstractness and concreteness are relative terms, and not a simple binary distinction.

This view is complemented by a suggestion about a significant role of social experience in acquisition and representation of abstract concepts (Barsalou and Wiemer-Hastings, 2005), since linguistic experience is acquired directly or indirectly in social interactions which makes it particularly crucial in building up abstract knowledge. Borghi et al. (2018) support this idea, considering words as social tools (WAT theory) and suggesting that abstract representations are more likely to involve linguistic and social experience than concrete ones (because of the absence of material references with objects), especially during their acquisition (Borghi and Binkofski, 2014; Borghi and Zarcone, 2016). WAT is an attempt to create an integral theory of abstract concepts from the point of embodied and grounded approach to cognition. We concur with Borghi et al. (2018)’ on the importance of exploring the differences between concrete and abstract concept acquisition but emphasize the need to focus on the dynamics of this acquisition process, not just on its outcomes.

## Neuroscientific Approaches

A different avenue for disentangling various accounts and interpretations of cognitive phenomena is offered in neuroscience, which focuses on identifying their underlying brain mechanisms, by investigating neuroanatomical substrates and neurophysiological dynamics of cognitive processes in the brain. In simple terms, if comprehension of concrete and abstract concepts is underpinned by different brain mechanisms, this can be investigated by scrutinizing neural activation patterns using functional brain mapping (e.g., EEG, MEG, fMRI or PET), or, to address causality, using neurostimulation techniques (TMS, tDCS) and/or brain-damaged patients. Neuropsychological data indicate that concrete words are more resistant to different brain injuries than abstract ones (Binder et al., 2005), suggesting at least partially different neural systems supporting these knowledge types. This suggestion is corroborated by a number of neuroscientific studies showing overlapping but not identical brain areas involved in abstract vs. concrete stimulus processing (see Montefinese, 2019, for a concise review).

However, there are still multiple contradictions across available neuroimaging studies (Wang et al., 2010), which has so far prevented neuroscience from resolving the dispute between theoretical accounts. Greater activation in such areas as middle and superior temporal gyrus (STG, MTG) and left inferior frontal gyrus (IFG) was associated with the processing of abstract concepts (Binder et al., 2005; Sabsevitz et al., 2005; Fliessbach et al., 2006; Pexman et al., 2007). Concrete concepts, in turn, have been shown to activate ventral anterior part of the fusiform gyrus (Sabsevitz et al., 2005; Bedny and Thompson-Schill, 2006; Fliessbach et al., 2006), which was also confirmed in an fMRI study of concrete word acquisition (Mestres-Misse et al., 2007). Other areas exhibit a less clear picture. For example, enhanced activation for abstract, as opposed to concrete, concepts has been observed in the anterior temporal region (ATL) in a number of studies (Tettamanti et al., 2008; Binder et al., 2009; Wang et al., 2010), whereas other experiments revealed the opposite, activation in ventral ATL specific for concrete concepts (Peelen and Caramazza, 2012; Visser et al., 2012; Robson et al., 2014), or an equal involvement of ventrolateral ATL for both concept types (Hoffman et al., 2015). It appears that while some such studies do not always have a clear basis in theoretical cognitive accounts, others mainly set out to prove the dual-coding theory. For instance, the results of EEG studies by Holcomb et al. (1999) speak in favor of the context-extended version of dual-coding account, which integrates DCT and CAT, at the neurophysiological level. Their experiments showed significant differences between brain responses to concrete and abstract words for the N400 component, a negative ERP wave associated with lexico-semantic processing: word concreteness leads to a greater negativity of the N400, especially in anterior areas, decreasing over posterior sites (Holcomb et al., 1999). Similar concreteness effect – stronger N400 – was also found in a study of acquisition of novel concrete and abstract semantics (Palmer et al., 2013). Concrete words also elicit larger N700 responses comparing with abstract ones even if they are matched for their context-availability and imageability (Barber et al., 2013), which, as the authors asserted, could not be explained by context-extended DCT. In turn, Pexman et al. (2007) unambiguously concluded that their neurophysiological data favors Barsalou’s theory of semantic representation over dual-coding and context-availability theories, while Borghi et al. (2018) find neurophysiological support for the WAT theory, further deepening the theoretical divide.

There are virtually no studies of concrete vs. abstract semantics using brain stimulation techniques (which could provide the much-needed causal evidence), with only a handful of TMS papers that suggested prefrontal and motor areas to take part in abstract word comprehension (e.g., Vukovic et al., 2017). One way to apply brain stimulation is to investigate changes in the activity of the motor cortex and corticospinal activation during comprehension (Hoffman et al., 2010). For example, the processing of abstract and concrete phrases differentially modulates cortico-spinal excitability (Scorolli et al., 2012). However, any association with movement will cause activation of the mirror neurons in the motor system (Rizzolatti and Sinigaglia, 2016), and given the great variability in motor cortex responses to TMS (Fedele et al., 2016), it is very difficult to disentangle the specific and non-specific effects of different semantic types and brain stimulation.

Clinical data distinguishing between abstract and concrete concepts are extremely rare. While there are some cases (“case studies”) of specific impairments in abstract or concrete concept comprehension, separately, they are based on very limited observations ranging from one to four patients at most (Warrington and Crutch, 2005; Crutch, 2006; Tree and Kay, 2006). Furthermore, such conceptual comprehension impairments are confounded by a variety of other co-morbidities (e.g., dyslexia in Crutch, 2006), while the definitions of abstractness and concreteness used by the authors vary and do not always conform to the status quo in the field. In essence, the available clinical data are so far unable to provide a clear picture of distinctions between these semantic types.

## Future Outlook

Whereas cognitive accounts of semantic representations, abstract semantics in particular, have gone a long way in recent decades, their neural counterparts so far suffer from the lack of studies and contradictions in the available data. The reasons for these contradictions could be many and include different properties of stimulus materials used, stimulation parameters, imaging modalities, and experimental tasks. One key difficulty lies with balancing basic psycholinguistic and physical properties of abstract and concrete words under investigations in a particular study; the lack of such balance confounds any differential results. A related issue that appears important is that most studies deal with pre-existing representations that are confounded by their surface properties, previous learning trajectories, daily use, and existing associations, all of which may obscure the results. In addition, the concrete-abstract dichotomy may not be complete and more fine-grain distinctions have been suggested: for example, action-related and object-/visually related concrete words, mental state-, emotion-, and mathematics-related abstract words (Dreyer and Pulvermüller, 2018). Further, rather than a dichotomy, there may be a multidimensional concreteness-abstractness continuum, along which words may vary, sometimes falling into both categories depending on the specific context (Myachykov and Fischer, 2019).

One way to circumvent these difficulties could be to assess the *process of acquisition* of novel concrete and abstract semantics in laboratory settings, using stimuli with fully controlled and systematically modulated semantic, physical and psycholinguistic parameters. By observing the learning process behaviorally and its counterparts in the brain, it may be possible to elucidate the systems that take part in building up novel representations and the degree to which they differ between semantic types. To avoid confounds related to different modes of acquisition of abstract and concrete semantics, the learning regime should be maximally matched between semantic conditions, using, for instance, context-based inference or direct instruction (Atir-Sharon et al., 2015). To assess the learning outcomes, an elaborate testing of lexical, semantic, and contextual levels of acquisition is desirable; ideally, the assessment should be done both immediately and after a consolidation period (e.g., after an overnight sleep, Davis et al., 2009).

Whereas many acquisition studies use either exceptionally novel word forms (pseudowords) (De Groot and Keijzer, 2000; Mestres-Missé et al., 2014) or unfamiliar words of foreign languages with established semantics (van Hell and Mahn, 1997), it is crucial to disentangle the mechanisms of learning the new word form and its phonology from those of acquiring the semantics *per se* (Partanen et al., 2017). This, in our view, is best achieved by training well-matched phonologically and phonotactically legal forms both as such (i.e., surface forms only) *and* in conjunction with novel semantics – rather than attaching familiar semantics to novel native word forms or foreign words (Leminen et al., 2016).

There is still a predominance of studies dedicated to investigation of learning mechanisms of concrete rather than abstract semantics; they are targeted more often owing to their more obvious link with sensorimotor experience (Mahon and Caramazza, 2008) that lends itself readily to experimental manipulation. While it may be straightforward to learn new names for new objects using, e.g., word-picture matching, creating a new abstract category in an experimental setting is much more challenging. One way to address this could be adopting abstract concepts from cultures other than that of experimental participants.

On another note, most available studies use correlational measures, e.g., showing distinct activation patterns accompanying perception. Clearly, causal evidence is also needed to demonstrate functional relevance of such distinctions. Outside of limited patient studies, such evidence is presently lacking. The use of neurostimulation techniques (such as TMS or tDCS) to influence both comprehension and acquisition of concrete and abstract semantics may provide the much-needed evidence for the involvement of particular brain areas in representing specific semantic types. For example, Fiori et al. (2011) revealed that the application of anodal tDCS over Wernicke’s area while learning new words significantly improved the accuracy and decreased latencies in a picture-naming task, while another study (Flöel et al., 2008) showed faster and better associative verbal learning with the anodal tDCS over posterior left perisylvian areas, compared to sham. We are not aware of any similar studies comparing concrete and abstract semantics and their acquisition; this could be the target for future investigations.

To conclude, the literature suggests cognitively and neurophysiologically distinct systems that support abstract and concrete representations in mind and brain. Yet, the data available to date, particularly with respect to abstract semantics, do not allow for clear delineation of the underlying brain systems and thus explaining the effects found behaviorally. To fill these gaps in the field, future studies should use a combination of rigorously matched behavioral regimes, controlled modes of presentation, a comprehensive set of tasks to assess behavioral outcomes at different times, and different neuroimaging tools able to assess both the complex dynamics of word comprehension and the causal relationships between brain structures and representation types. One way to help disentangle the mechanisms underpinning different semantic representations is to focus on their acquisition in controlled experimental settings.

## Author Contributions

All authors listed have made a substantial, direct and intellectual contribution to the work, and approved it for publication.

## Conflict of Interest Statement

The authors declare that the research was conducted in the absence of any commercial or financial relationships that could be construed as a potential conflict of interest.
